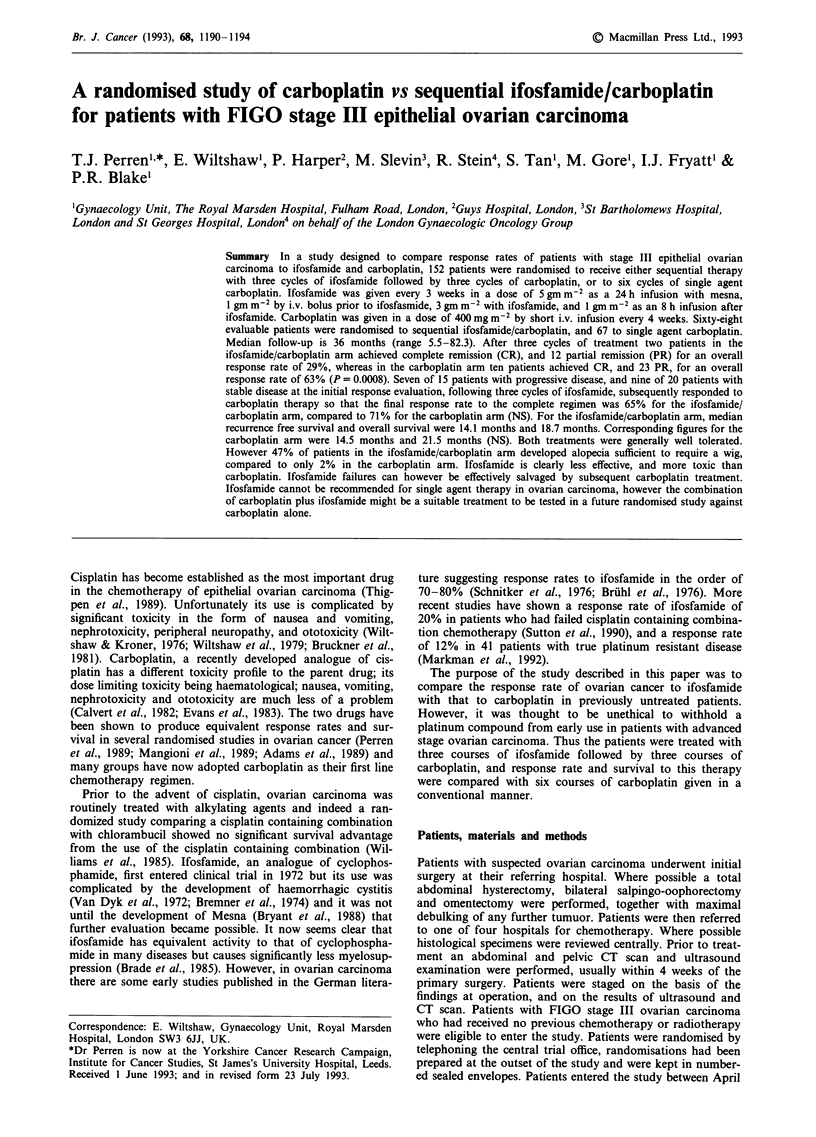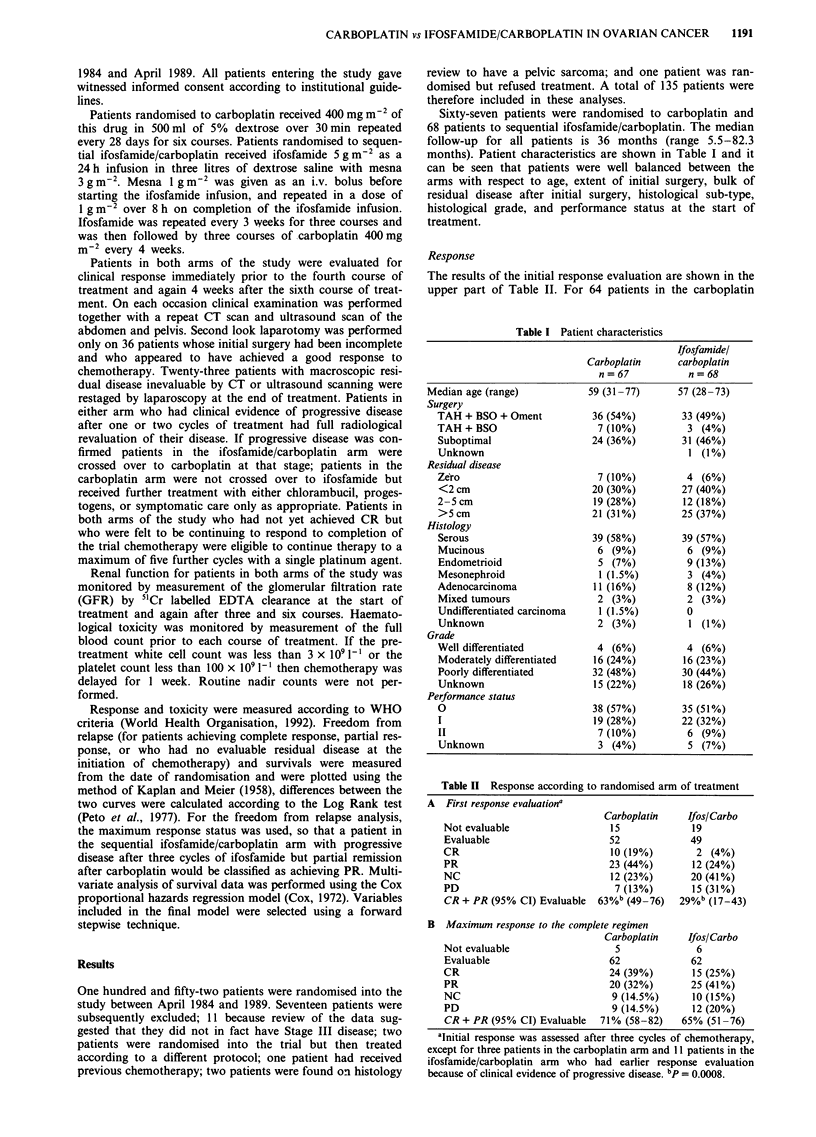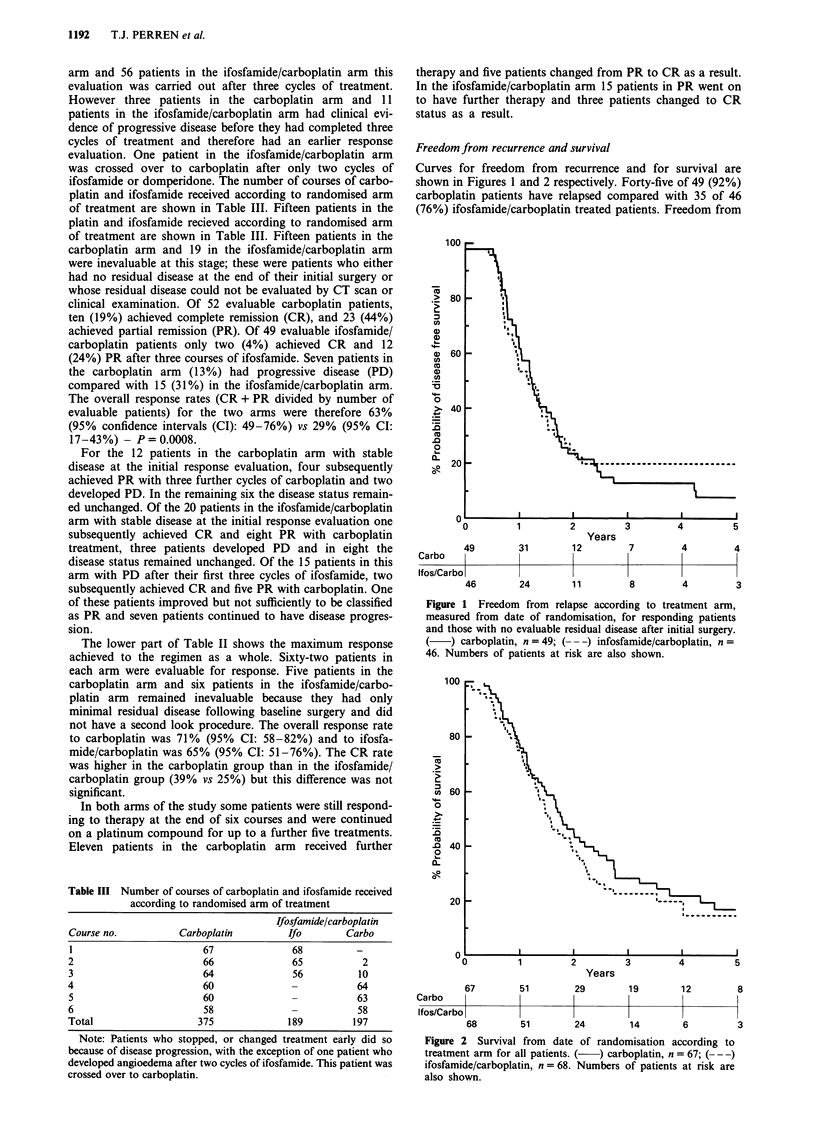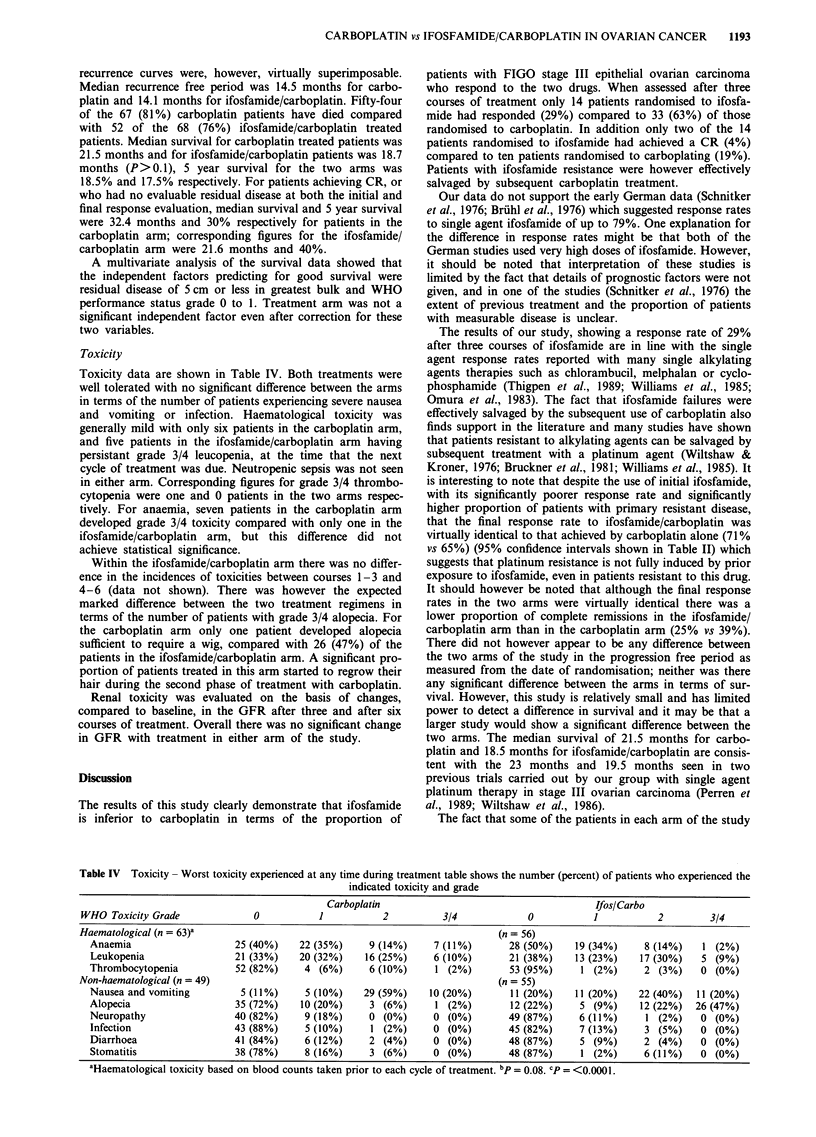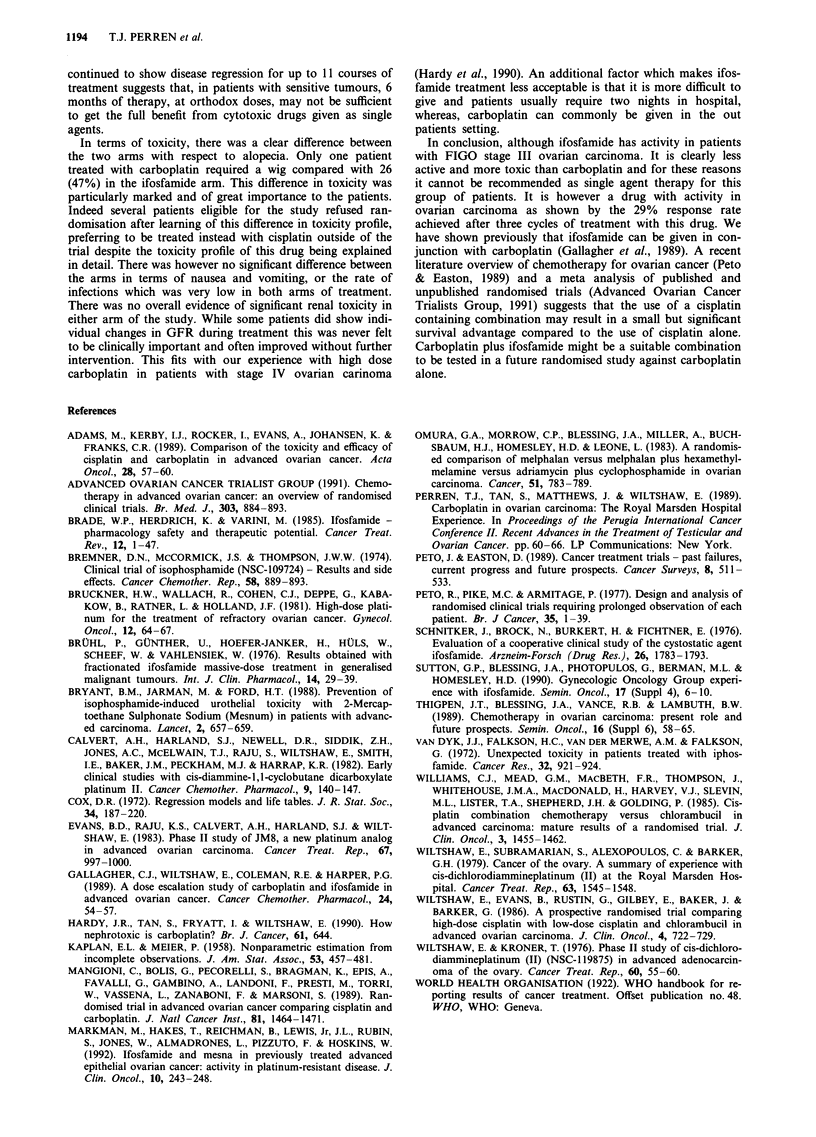# A randomised study of carboplatin vs sequential ifosfamide/carboplatin for patients with FIGO stage III epithelial ovarian carcinoma. The London Gynaecologic Oncology Group.

**DOI:** 10.1038/bjc.1993.502

**Published:** 1993-12

**Authors:** T. J. Perren, E. Wiltshaw, P. Harper, M. Slevin, R. Stein, S. Tan, M. Gore, I. J. Fryatt, P. R. Blake

**Affiliations:** Gynaecology Unit, Royal Marsden Hospital, London, UK.

## Abstract

In a study designed to compare response rates of patients with stage III epithelial ovarian carcinoma to ifosfamide and carboplatin, 152 patients were randomised to receive either sequential therapy with three cycles of ifosfamide followed by three cycles of carboplatin, or to six cycles of single agent carboplatin. Ifosfamide was given every 3 weeks in a dose of 5 gm m-2 as a 24 h infusion with mesna, 1 gm m-2 by i.v. bolus prior to ifosfamide, 3 gm m-2 with ifosfamide, and 1 gm m-2 as an 8 h infusion after ifosfamide. Carboplatin was given in a dose of 400 mg m-2 by short i.v. infusion every 4 weeks. Sixty-eight evaluable patients were randomised to sequential ifosfamide/carboplatin, and 67 to single agent carboplatin. Median follow-up is 36 months (range 5.5-82.3). After three cycles of treatment two patients in the ifosfamide/carboplatin arm achieved complete remission (CR), and 12 partial remission (PR) for an overall response rate of 29%, whereas in the carboplatin arm ten patients achieved CR, and 23 PR, for an overall response rate of 63% (P = 0.0008). Seven of 15 patients with progressive disease, and nine of 20 patients with stable disease at the initial response evaluation, following three cycles of ifosfamide, subsequently responded to carboplatin therapy so that the final response rate to the complete regimen was 65% for the ifosfamide/carboplatin arm, compared to 71% for the carboplatin arm (NS). For the ifosfamide/carboplatin arm, median recurrence free survival and overall survival were 14.1 months and 18.7 months. Corresponding figures for the carboplatin arm were 14.5 months and 21.5 months (NS). Both treatments were generally well tolerated. However 47% of patients in the ifosfamide/carboplatin arm developed alopecia sufficient to require a wig, compared to only 2% in the carboplatin arm. Ifosfamide is clearly less effective, and more toxic than carboplatin. Ifosfamide failures can however be effectively salvaged by subsequent carboplatin treatment. Ifosfamide cannot be recommended for single agent therapy in ovarian carcinoma, however the combination of carboplatin plus ifosfamide might be a suitable treatment to be tested in a future randomised study against carboplatin alone.


Br. J. Cancer (1993), 68, 1190–1194 © Macmillan Press Ltd., 1993

### A randomised study of carboplatin *vs* sequential ifosfamide/carboplatin for patients with FIGO stage III epithelial ovarian carcinoma

T.J. Perren[1,*], E. Wiltshaw[1], P. Harper[2], M. Slevin[3], R. Stein[4], S. Tan[1], M. Gore[1], I.J. Fryatt[1] & P.R. Blake[1]

[1]*Gynaecology Unit, The Royal Marsden Hospital, Fulham Road, London,* [2]*Guys Hospital, London,* [3]*St Bartholomews Hospital, London and St Georges Hospital, London*[4] *on behalf of the London Gynaecologic Oncology Group*

**Summary** In a study designed to compare response rates of patients with stage III epithelial ovarian carcinoma to ifosfamide and carboplatin, 152 patients were randomised to receive either sequential therapy with three cycles of ifosfamide followed by three cycles of carboplatin, or to six cycles of single agent carboplatin. Ifosfamide was given every 3 weeks in a dose of 5 gm m$^{-2}$ as a 24 h infusion with mesna, 1 gm m$^{-2}$ by i.v. bolus prior to ifosfasmide, 3 gm m$^{-2}$ with ifosfamide, and 1 gm m$^{-2}$ as an 8 h infusion after ifosfamide. Carboplatin was given in a dose of 400 mg m$^{-2}$ by short i.v. infusion every 4 weeks. Sixty-eight evaluable patients were randomised to sequential ifosfamide/carboplatin, and 67 to single agent carboplatin. Median follow-up is 36 months (range 5.5–82.3). After three cycles of treatment two patients in the ifosfamide/carboplatin arm achieved complete remission (CR), and 12 partial remission (PR) for an overall response rate of 29%, whereas in the carboplatin arm ten patients achieved CR, and 23 PR, for an overall response rate of 63% ($P = 0.0008$). Seven of 15 patients with progressive disease, and nine of 20 patients with stable disease at the initial response evaluation, following three cycles of ifosfamide, subsequently responded to carboplatin therapy so that the final response rate to the complete regimen was 65% for the ifosfamide/carboplatin arm, compared to 71% for the carboplatin arm (NS). For the ifosfamide/carboplatin arm, median recurrence free survival and overall survival were 14.1 months and 18.7 months. Corresponding figures for the carboplatin arm were 14.5 months and 21.5 months (NS). Both treatments were generally well tolerated. However 47% of patients in the ifosfamide/carboplatin arm developed alopecia sufficient to require a wig, compared to only 2% in the carboplatin arm. Ifosfamide is clearly less effective, and more toxic than carboplatin. Ifosfamide failures can however be effectively salvaged by subsequent carboplatin treatment. Ifosfamide cannot be recommended for single agent therapy in ovarian carcinoma, however the combination of carboplatin plus ifosfamide might be a suitable treatment to be tested in a future randomised study against carboplatin alone.

Cisplatin has become established as the most important drug in the chemotherapy of epithelial ovarian carcinoma (Thigpen *et al.*, 1989). Unfortunately its use is complicated by significant toxicity in the form of nausea and vomiting, nephrotoxicity, peripheral neuropathy, and ototoxicity (Wiltshaw & Kroner, 1976; Wiltshaw *et al.*, 1979; Bruckner *et al.*, 1981). Carboplatin, a recently developed analogue of cisplatin has a different toxicity profile to the parent drug; its dose limiting toxicity being haematological; nausea, vomiting, nephrotoxicity and ototoxicity are much less of a problem (Calvert *et al.*, 1982; Evans *et al.*, 1983). The two drugs have been shown to produce equivalent response rates and survival in several randomised studies in ovarian cancer (Perren *et al.*, 1989; Mangioni *et al.*, 1989; Adams *et al.*, 1989) and many groups have now adopted carboplatin as their first line chemotherapy regimen.

Prior to the advent of cisplatin, ovarian carcinoma was routinely treated with alkylating agents and indeed a randomized study comparing a cisplatin containing combination with chlorambucil showed no significant survival advantage from the use of the cisplatin containing combination (Williams *et al.*, 1985). Ifosfamide, an analogue of cyclophosphamide, first entered clinical trial in 1972 but its use was complicated by the development of haemorrhagic cystitis (Van Dyk *et al.*, 1972; Bremner *et al.*, 1974) and it was not until the development of Mesna (Bryant *et al.*, 1988) that further evaluation became possible. It now seems clear that ifosfamide has equivalent activity to that of cyclophosphamide in many diseases but causes significantly less myelosuppression (Brade *et al.*, 1985). However, in ovarian carcinoma there are some early studies published in the German literature suggesting response rates to ifosfamide in the order of 70–80% (Schnitker *et al.*, 1976; Brühl *et al.*, 1976). More recent studies have shown a response rate of ifosfamide of 20% in patients who had failed cisplatin containing combination chemotherapy (Sutton *et al.*, 1990), and a response rate of 12% in 41 patients with true platinum resistant disease (Markman *et al.*, 1992).

The purpose of the study described in this paper was to compare the response rate of ovarian cancer to ifosfamide with that to carboplatin in previously untreated patients. However, it was thought to be unethical to withhold a platinum compound from early use in patients with advanced stage ovarian carcinoma. Thus the patients were treated with three courses of ifosfamide followed by three courses of carboplatin, and response rate and survival to this therapy were compared with six courses of carboplatin given in a conventional manner.

### Patients, materials and methods

Patients with suspected ovarian carcinoma underwent initial surgery at their referring hospital. Where possible a total abdominal hysterectomy, bilateral salpingo-oophorectomy and omentectomy were performed, together with maximal debulking of any further tumuor. Patients were then referred to one of four hospitals for chemotherapy. Where possible histological specimens were reviewed centrally. Prior to treatment an abdominal and pelvic CT scan and ultrasound examination were performed, usually within 4 weeks of the primary surgery. Patients were staged on the basis of the findings at operation, and on the results of ultrasound and CT scan. Patients with FIGO stage III ovarian carcinoma who had received no previous chemotherapy or radiotherapy were eligible to enter the study. Patients were randomised by telephoning the central trial office, randomisations had been prepared at the outset of the study and were kept in numbered sealed envelopes. Patients entered the study between April

Correspondence: E. Wiltshaw, Gynaecology Unit, Royal Marsden Hospital, London SW3 6JJ, UK.
*Dr Perren is now at the Yorkshire Cancer Research Campaign, Institute for Cancer Studies, St James's University Hospital, Leeds.
Received 1 June 1993; and in revised form 23 July 1993.

1984 and April 1989. All patients entering the study gave witnessed informed consent according to institutional guidelines.

Patients randomised to carboplatin received 400 mg $m^{-2}$ of this drug in 500 ml of 5% dextrose over 30 min repeated every 28 days for six courses. Patients randomised to sequential ifosfamide/carboplatin received ifosfamide 5 g $m^{-2}$ as a 24 h infusion in three litres of dextrose saline with mesna 3 g $m^{-2}$. Mesna 1 g $m^{-2}$ was given as an i.v. bolus before starting the ifosfamide infusion, and repeated in a dose of 1 g $m^{-2}$ over 8 h on completion of the ifosfamide infusion. Ifosfamide was repeated every 3 weeks for three courses and was then followed by three courses of carboplatin 400 mg $m^{-2}$ every 4 weeks.

Patients in both arms of the study were evaluated for clinical response immediately prior to the fourth course of treatment and again 4 weeks after the sixth course of treatment. On each occasion clinical examination was performed together with a repeat CT scan and ultrasound scan of the abdomen and pelvis. Second look laparotomy was performed only on 36 patients whose initial surgery had been incomplete and who appeared to have achieved a good response to chemotherapy. Twenty-three patients with macroscopic residual disease inevaluable by CT or ultrasound scanning were restaged by laparoscopy at the end of treatment. Patients in either arm who had clinical evidence of progressive disease after one or two cycles of treatment had full radiological revaluation of their disease. If progressive disease was confirmed patients in the ifosfamide/carboplatin arm were crossed over to carboplatin at that stage; patients in the carboplatin arm were not crossed over to ifosfamide but received further treatment with either chlorambucil, progestogens, or symptomatic care only as appropriate. Patients in both arms of the study who had not yet achieved CR but who were felt to be continuing to respond to completion of the trial chemotherapy were eligible to continue therapy to a maximum of five further cycles with a single platinum agent.

Renal function for patients in both arms of the study was monitored by measurement of the glomerular filtration rate (GFR) by $^{51}Cr$ labelled EDTA clearance at the start of treatment and again after three and six courses. Haematological toxicity was monitored by measurement of the full blood count prior to each course of treatment. If the pretreatment white cell count was less than $3 \times 10^9 l^{-1}$ or the platelet count less than $100 \times 10^9 l^{-1}$ then chemotherapy was delayed for 1 week. Routine nadir counts were not performed.

Response and toxicity were measured according to WHO criteria (World Health Organisation, 1992). Freedom from relapse (for patients achieving complete response, partial response, or who had no evaluable residual disease at the initiation of chemotherapy) and survivals were measured from the date of randomisation and were plotted using the method of Kaplan and Meier (1958), differences between the two curves were calculated according to the Log Rank test (Peto *et al.*, 1977). For the freedom from relapse analysis, the maximum response status was used, so that a patient in the sequential ifosfamide/carboplatin arm with progressive disease after three cycles of ifosfamide but partial remission after carboplatin would be classified as achieving PR. Multivariate analysis of survival data was performed using the Cox proportional hazards regression model (Cox, 1972). Variables included in the final model were selected using a forward stepwise technique.

### Results

One hundred and fifty-two patients were randomised into the study between April 1984 and 1989. Seventeen patients were subsequently excluded; 11 because review of the data suggested that they did not in fact have Stage III disease; two patients were randomised into the trial but then treated according to a different protocol; one patient had received previous chemotherapy; two patients were found on histology review to have a pelvic sarcoma; and one patient was randomised but refused treatment. A total of 135 patients were therefore included in these analyses.

Sixty-seven patients were randomised to carboplatin and 68 patients to sequential ifosfamide/carboplatin. The median follow-up for all patients is 36 months (range 5.5–82.3 months). Patient characteristics are shown in Table I and it can be seen that patients were well balanced between the arms with respect to age, extent of initial surgery, bulk of residual disease after initial surgery, histological sub-type, histological grade, and performance status at the start of treatment.

### *Response*

The results of the initial response evaluation are shown in the upper part of Table II. For 64 patients in the carboplatin

**Table I** Patient characteristics

| | *Carboplatin* *n* = 67 | *Ifosfamide/ carboplatin* *n* = 68 |
|---|---|---|
| Median age (range) | 59 (31–77) | 57 (28–73) |
| *Surgery* | | |
| TAH + BSO + Oment | 36 (54%) | 33 (49%) |
| TAH + BSO | 7 (10%) | 3 (4%) |
| Suboptimal | 24 (36%) | 31 (46%) |
| Unknown | | 1 (1%) |
| *Residual disease* | | |
| Zero | 7 (10%) | 4 (6%) |
| <2 cm | 20 (30%) | 27 (40%) |
| 2–5 cm | 19 (28%) | 12 (18%) |
| >5 cm | 21 (31%) | 25 (37%) |
| *Histology* | | |
| Serous | 39 (58%) | 39 (57%) |
| Mucinous | 6 (9%) | 6 (9%) |
| Endometrioid | 5 (7%) | 9 (13%) |
| Mesonephroid | 1 (1.5%) | 3 (4%) |
| Adenocarcinoma | 11 (16%) | 8 (12%) |
| Mixed tumours | 2 (3%) | 2 (3%) |
| Undifferentiated carcinoma | 1 (1.5%) | 0 |
| Unknown | 2 (3%) | 1 (1%) |
| *Grade* | | |
| Well differentiated | 4 (6%) | 4 (6%) |
| Moderately differentiated | 16 (24%) | 16 (23%) |
| Poorly differentiated | 32 (48%) | 30 (44%) |
| Unknown | 15 (22%) | 18 (26%) |
| *Performance status* | | |
| O | 38 (57%) | 35 (51%) |
| I | 19 (28%) | 22 (32%) |
| II | 7 (10%) | 6 (9%) |
| Unknown | 3 (4%) | 5 (7%) |

**Table II** Response according to randomised arm of treatment

| | *Carboplatin* | *Ifos/Carbo* |
|---|---|---|
| **A** *First response evaluation*[a] | | |
| Not evaluable | 15 | 19 |
| Evaluable | 52 | 49 |
| CR | 10 (19%) | 2 (4%) |
| PR | 23 (44%) | 12 (24%) |
| NC | 12 (23%) | 20 (41%) |
| PD | 7 (13%) | 15 (31%) |
| *CR* + *PR* (95% CI) Evaluable | 63%[b] (49–76) | 29%[b] (17–43) |
| **B** *Maximum response to the complete regimen* | *Carboplatin* | *Ifos/Carbo* |
| Not evaluable | 5 | 6 |
| Evaluable | 62 | 62 |
| CR | 24 (39%) | 15 (25%) |
| PR | 20 (32%) | 25 (41%) |
| NC | 9 (14.5%) | 10 (15%) |
| PD | 9 (14.5%) | 12 (20%) |
| *CR* + *PR* (95% CI) Evaluable | 71% (58–82) | 65% (51–76) |

[a]Initial response was assessed after three cycles of chemotherapy, except for three patients in the carboplatin arm and 11 patients in the ifosfamide/carboplatin arm who had earlier response evaluation because of clinical evidence of progressive disease. [b]$P = 0.0008$.

arm and 56 patients in the ifosfamide/carboplatin arm this evaluation was carried out after three cycles of treatment. However three patients in the carboplatin arm and 11 patients in the ifosfamide/carboplatin arm had clinical evidence of progressive disease before they had completed three cycles of treatment and therefore had an earlier response evaluation. One patient in the ifosfamide/carboplatin arm was crossed over to carboplatin after only two cycles of ifosfamide or domperidone. The number of courses of carboplatin and ifosfamide received according to randomised arm of treatment are shown in Table III. Fifteen patients in the platin and ifosfamide recieved according to randomised arm of treatment are shown in Table III. Fifteen patients in the carboplatin arm and 19 in the ifosfamide/carboplatin arm were inevaluable at this stage; these were patients who either had no residual disease at the end of their initial surgery or whose residual disease could not be evaluated by CT scan or clinical examination. Of 52 evaluable carboplatin patients, ten (19%) achieved complete remission (CR), and 23 (44%) achieved partial remission (PR). Of 49 evaluable ifosfamide/carboplatin patients only two (4%) achieved CR and 12 (24%) PR after three courses of ifosfamide. Seven patients in the carboplatin arm (13%) had progressive disease (PD) compared with 15 (31%) in the ifosfamide/carboplatin arm. The overall response rates (CR + PR divided by number of evaluable patients) for the two arms were therefore 63% (95% confidence intervals (CI): 49–76%) *vs* 29% (95% CI: 17–43%) – $P = 0.0008$.

For the 12 patients in the carboplatin arm with stable disease at the initial response evaluation, four subsequently achieved PR with three further cycles of carboplatin and two developed PD. In the remaining six the disease status remained unchanged. Of the 20 patients in the ifosfamide/carboplatin arm with stable disease at the initial response evaluation one subsequently achieved CR and eight PR with carboplatin treatment, three patients developed PD and in eight the disease status remained unchanged. Of the 15 patients in this arm with PD after their first three cycles of ifosfamide, two subsequently achieved CR and five PR with carboplatin. One of these patients improved but not sufficiently to be classified as PR and seven patients continued to have disease progression.

The lower part of Table II shows the maximum response achieved to the regimen as a whole. Sixty-two patients in each arm were evaluable for response. Five patients in the carboplatin arm and six patients in the ifosfamide/carboplatin arm remained inevaluable because they had only minimal residual disease following baseline surgery and did not have a second look procedure. The overall response rate to carboplatin was 71% (95% CI: 58–82%) and to ifosfamide/carboplatin was 65% (95% CI: 51–76%). The CR rate was higher in the carboplatin group than in the ifosfamide/carboplatin group (39% *vs* 25%) but this difference was not significant.

In both arms of the study some patients were still responding to therapy at the end of six courses and were continued on a platinum compound for up to a further five treatments. Eleven patients in the carboplatin arm received further

**Table III** Number of courses of carboplatin and ifosfamide received according to randomised arm of treatment

| | | Ifosfamide/carboplatin | |
|---|---|---|---|
| *Course no.* | *Carboplatin* | *Ifo* | *Carbo* |
| 1 | 67 | 68 | – |
| 2 | 66 | 65 | 2 |
| 3 | 64 | 56 | 10 |
| 4 | 60 | – | 64 |
| 5 | 60 | – | 63 |
| 6 | 58 | – | 58 |
| Total | 375 | 189 | 197 |

Note: Patients who stopped, or changed treatment early did so because of disease progression, with the exception of one patient who developed angioedema after two cycles of ifosfamide. This patient was crossed over to carboplatin.

therapy and five patients changed from PR to CR as a result. In the ifosfamide/carboplatin arm 15 patients in PR went on to have further therapy and three patients changed to CR status as a result.

*Freedom from recurrence and survival*

Curves for freedom from recurrence and for survival are shown in Figures 1 and 2 respectively. Forty-five of 49 (92%) carboplatin patients have relapsed compared with 35 of 46 (76%) ifosfamide/carboplatin treated patients. Freedom from

**Figure 1** Freedom from relapse according to treatment arm, measured from date of randomisation, for responding patients and those with no evaluable residual disease after initial surgery. (——) carboplatin, $n = 49$; (- - -) infosfamide/carboplatin, $n = 46$. Numbers of patients at risk are also shown.

**Figure 2** Survival from date of randomisation according to treatment arm for all patients. (——) carboplatin, $n = 67$; (- - -) ifosfamide/carboplatin, $n = 68$. Numbers of patients at risk are also shown.

recurrence curves were, however, virtually superimposable. Median recurrence free period was 14.5 months for carboplatin and 14.1 months for ifosfamide/carboplatin. Fifty-four of the 67 (81%) carboplatin patients have died compared with 52 of the 68 (76%) ifosfamide/carboplatin treated patients. Median survival for carboplatin treated patients was 21.5 months and for ifosfamide/carboplatin patients was 18.7 months ($P > 0.1$), 5 year survival for the two arms was 18.5% and 17.5% respectively. For patients achieving CR, or who had no evaluable residual disease at both the initial and final response evaluation, median survival and 5 year survival were 32.4 months and 30% respectively for patients in the carboplatin arm; corresponding figures for the ifosfamide/carboplatin arm were 21.6 months and 40%.

A multivariate analysis of the survival data showed that the independent factors predicting for good survival were residual disease of 5 cm or less in greatest bulk and WHO performance status grade 0 to 1. Treatment arm was not a significant independent factor even after correction for these two variables.

### *Toxicity*

Toxicity data are shown in Table IV. Both treatments were well tolerated with no significant difference between the arms in terms of the number of patients experiencing severe nausea and vomiting or infection. Haematological toxicity was generally mild with only six patients in the carboplatin arm, and five patients in the ifosfamide/carboplatin arm having persistant grade 3/4 leucopenia, at the time that the next cycle of treatment was due. Neutropenic sepsis was not seen in either arm. Corresponding figures for grade 3/4 thrombocytopenia were one and 0 patients in the two arms respectively. For anaemia, seven patients in the carboplatin arm developed grade 3/4 toxicity compared with only one in the ifosfamide/carboplatin arm, but this difference did not achieve statistical significance.

Within the ifosfamide/carboplatin arm there was no difference in the incidences of toxicities between courses 1–3 and 4–6 (data not shown). There was however the expected marked difference between the two treatment regimens in terms of the number of patients with grade 3/4 alopecia. For the carboplatin arm only one patient developed alopecia sufficient to require a wig, compared with 26 (47%) of the patients in the ifosfamide/carboplatin arm. A significant proportion of patients treated in this arm started to regrow their hair during the second phase of treatment with carboplatin.

Renal toxicity was evaluated on the basis of changes, compared to baseline, in the GFR after three and after six courses of treatment. Overall there was no significant change in GFR with treatment in either arm of the study.

### Discussion

The results of this study clearly demonstrate that ifosfamide is inferior to carboplatin in terms of the proportion of patients with FIGO stage III epithelial ovarian carcinoma who respond to the two drugs. When assessed after three courses of treatment only 14 patients randomised to ifosfamide had responded (29%) compared to 33 (63%) of those randomised to carboplatin. In addition only two of the 14 patients randomised to ifosfamide had achieved a CR (4%) compared to ten patients randomised to carboplating (19%). Patients with ifosfamide resistance were however effectively salvaged by subsequent carboplatin treatment.

Our data do not support the early German data (Schnitker *et al.*, 1976; Brühl *et al.*, 1976) which suggested response rates to single agent ifosfamide of up to 79%. One explanation for the difference in response rates might be that both of the German studies used very high doses of ifosfamide. However, it should be noted that interpretation of these studies is limited by the fact that details of prognostic factors were not given, and in one of the studies (Schnitker *et al.*, 1976) the extent of previous treatment and the proportion of patients with measurable disease is unclear.

The results of our study, showing a response rate of 29% after three courses of ifosfamide are in line with the single agent response rates reported with many single alkylating agents therapies such as chlorambucil, melphalan or cyclophosphamide (Thigpen *et al.*, 1989; Williams *et al.*, 1985; Omura *et al.*, 1983). The fact that ifosfamide failures were effectively salvaged by the subsequent use of carboplatin also finds support in the literature and many studies have shown that patients resistant to alkylating agents can be salvaged by subsequent treatment with a platinum agent (Wiltshaw & Kroner, 1976; Bruckner *et al.*, 1981; Williams *et al.*, 1985). It is interesting to note that despite the use of initial ifosfamide, with its significantly poorer response rate and significantly higher proportion of patients with primary resistant disease, that the final response rate to ifosfamide/carboplatin was virtually identical to that achieved by carboplatin alone (71% *vs* 65%) (95% confidence intervals shown in Table II) which suggests that platinum resistance is not fully induced by prior exposure to ifosfamide, even in patients resistant to this drug. It should however be noted that although the final response rates in the two arms were virtually identical there was a lower proportion of complete remissions in the ifosfamide/carboplatin arm than in the carboplatin arm (25% *vs* 39%). There did not however appear to be any difference between the two arms of the study in the progression free period as measured from the date of randomisation; neither was there any significant difference between the arms in terms of survival. However, this study is relatively small and has limited power to detect a difference in survival and it may be that a larger study would show a significant difference between the two arms. The median survival of 21.5 months for carboplatin and 18.5 months for ifosfamide/carboplatin are consistent with the 23 months and 19.5 months seen in two previous trials carried out by our group with single agent platinum therapy in stage III ovarian carcinoma (Perren *et al.*, 1989; Wiltshaw *et al.*, 1986).

The fact that some of the patients in each arm of the study

**Table IV** Toxicity – Worst toxicity experienced at any time during treatment table shows the number (percent) of patients who experienced the indicated toxicity and grade

| | *Carboplatin* | | | | *Ifos/Carbo* | | | |
|---|---|---|---|---|---|---|---|---|
| *WHO Toxicity Grade* | *0* | *1* | *2* | *3/4* | *0* | *1* | *2* | *3/4* |
| *Haematological* ($n = 63$)[a] | | | | | ($n = 56$) | | | |
| Anaemia | 25 (40%) | 22 (35%) | 9 (14%) | 7 (11%) | 28 (50%) | 19 (34%) | 8 (14%) | 1 (2%) |
| Leukopenia | 21 (33%) | 20 (32%) | 16 (25%) | 6 (10%) | 21 (38%) | 13 (23%) | 17 (30%) | 5 (9%) |
| Thrombocytopenia | 52 (82%) | 4 (6%) | 6 (10%) | 1 (2%) | 53 (95%) | 1 (2%) | 2 (3%) | 0 (0%) |
| *Non-haematological* ($n = 49$) | | | | | ($n = 55$) | | | |
| Nausea and vomiting | 5 (11%) | 5 (10%) | 29 (59%) | 10 (20%) | 11 (20%) | 11 (20%) | 22 (40%) | 11 (20%) |
| Alopecia | 35 (72%) | 10 (20%) | 3 (6%) | 1 (2%) | 12 (22%) | 5 (9%) | 12 (22%) | 26 (47%) |
| Neuropathy | 40 (82%) | 9 (18%) | 0 (0%) | 0 (0%) | 49 (87%) | 6 (11%) | 1 (2%) | 0 (0%) |
| Infection | 43 (88%) | 5 (10%) | 1 (2%) | 0 (0%) | 45 (82%) | 7 (13%) | 3 (5%) | 0 (0%) |
| Diarrhoea | 41 (84%) | 6 (12%) | 2 (4%) | 0 (0%) | 48 (87%) | 5 (9%) | 2 (4%) | 0 (0%) |
| Stomatitis | 38 (78%) | 8 (16%) | 3 (6%) | 0 (0%) | 48 (87%) | 1 (2%) | 6 (11%) | 0 (0%) |

[a]Haematological toxicity based on blood counts taken prior to each cycle of treatment. [b]$P = 0.08$. [c]$P = < 0.0001$.

continued to show disease regression for up to 11 courses of treatment suggests that, in patients with sensitive tumours, 6 months of therapy, at orthodox doses, may not be sufficient to get the full benefit from cytotoxic drugs given as single agents.

In terms of toxicity, there was a clear difference between the two arms with respect to alopecia. Only one patient treated with carboplatin required a wig compared with 26 (47%) in the ifosfamide arm. This difference in toxicity was particularly marked and of great importance to the patients. Indeed several patients eligible for the study refused randomisation after learning of this difference in toxicity profile, preferring to be treated instead with cisplatin outside of the trial despite the toxicity profile of this drug being explained in detail. There was however no significant difference between the arms in terms of nausea and vomiting, or the rate of infections which was very low in both arms of treatment. There was no overall evidence of significant renal toxicity in either arm of the study. While some patients did show individual changes in GFR during treatment this was never felt to be clinically important and often improved without further intervention. This fits with our experience with high dose carboplatin in patients with stage IV ovarian carinoma (Hardy *et al.*, 1990). An additional factor which makes ifosfamide treatment less acceptable is that it is more difficult to give and patients usually require two nights in hospital, whereas, carboplatin can commonly be given in the out patients setting.

In conclusion, although ifosfamide has activity in patients with FIGO stage III ovarian carcinoma. It is clearly less active and more toxic than carboplatin and for these reasons it cannot be recommended as single agent therapy for this group of patients. It is however a drug with activity in ovarian carcinoma as shown by the 29% response rate achieved after three cycles of treatment with this drug. We have shown previously that ifosfamide can be given in conjunction with carboplatin (Gallagher *et al.*, 1989). A recent literature overview of chemotherapy for ovarian cancer (Peto & Easton, 1989) and a meta analysis of published and unpublished randomised trials (Advanced Ovarian Cancer Trialists Group, 1991) suggests that the use of a cisplatin containing combination may result in a small but significant survival advantage compared to the use of cisplatin alone. Carboplatin plus ifosfamide might be a suitable combination to be tested in a future randomised study against carboplatin alone.

### References

ADAMS, M., KERBY, I.J., ROCKER, I., EVANS, A., JOHANSEN, K. & FRANKS, C.R. (1989). Comparison of the toxicity and efficacy of cisplatin and carboplatin in advanced ovarian cancer. *Acta Oncol.*, **28**, 57-60.

ADVANCED OVARIAN CANCER TRIALIST GROUP (1991). Chemotherapy in advanced ovarian cancer: an overview of randomised clinical trials. *Br. Med. J.*, **303**, 884-893.

BRADE, W.P., HERDRICH, K. & VARINI, M. (1985). Ifosfamide – pharmacology safety and therapeutic potential. *Cancer Treat. Rev.*, **12**, 1-47.

BREMNER, D.N., MCCORMICK, J.S. & THOMPSON, J.W.W. (1974). Clinical trial of isophosphamide (NSC-109724) – Results and side effects. *Cancer Chemother. Rep.*, **58**, 889-893.

BRUCKNER, H.W., WALLACH, R., COHEN, C.J., DEPPE, G., KABAKOW, B., RATNER, L. & HOLLAND, J.F. (1981). High-dose platinum for the treatment of refractory ovarian cancer. *Gynecol. Oncol.*, **12**, 64-67.

BRÜHL, P., GÜNTHER, U., HOEFER-JANKER, H., HÜLS, W., SCHEEF, W. & VAHLENSIEK, W. (1976). Results obtained with fractionated ifosfamide massive-dose treatment in generalised malignant tumours. *Int. J. Clin. Pharmacol.*, **14**, 29-39.

BRYANT, B.M., JARMAN, M. & FORD, H.T. (1988). Prevention of isophosphamide-induced urothelial toxicity with 2-Mercaptoethane Sulphonate Sodium (Mesnum) in patients with advanced carcinoma. *Lancet*, **2**, 657-659.

CALVERT, A.H., HARLAND, S.J., NEWELL, D.R., SIDDIK, Z.H., JONES, A.C., MCELWAIN, T.J., RAJU, S., WILTSHAW, E., SMITH, I.E., BAKER, J.M., PECKHAM, M.J. & HARRAP, K.R. (1982). Early clinical studies with cis-diammine-1,1-cyclobutane dicarboxylate platinum II. *Cancer Chemother. Pharmacol.*, **9**, 140-147.

COX, D.R. (1972). Regression models and life tables. *J. R. Stat. Soc.*, **34**, 187-220.

EVANS, B.D., RAJU, K.S., CALVERT, A.H., HARLAND, S.J. & WILTSHAW, E. (1983). Phase II study of JM8, a new platinum analog in advanced ovarian carcinoma. *Cancer Treat. Rep.*, **67**, 997-1000.

GALLAGHER, C.J., WILTSHAW, E., COLEMAN, R.E. & HARPER, P.G. (1989). A dose escalation study of carboplatin and ifosfamide in advanced ovarian cancer. *Cancer Chemother. Pharmacol.*, **24**, 54-57.

HARDY, J.R., TAN, S., FRYATT, I. & WILTSHAW, E. (1990). How nephrotoxic is carboplatin? *Br. J. Cancer*, **61**, 644.

KAPLAN, E.L. & MEIER, P. (1958). Nonparametric estimation from incomplete observations. *J. Am. Stat. Assoc.*, **53**, 457-481.

MANGIONI, C., BOLIS, G., PECORELLI, S., BRAGMAN, K., EPIS, A., FAVALLI, G., GAMBINO, A., LANDONI, F., PRESTI, M., TORRI, W., VASSENA, L., ZANABONI, F. & MARSONI, S. (1989). Randomised trial in advanced ovarian cancer comparing cisplatin and carboplatin. *J. Natl Cancer Inst.*, **81**, 1464-1471.

MARKMAN, M., HAKES, T., REICHMAN, B., LEWIS, Jr, J.L., RUBIN, S., JONES, W., ALMADRONES, L., PIZZUTO, F. & HOSKINS, W. (1992). Ifosfamide and mesna in previously treated advanced epithelial ovarian cancer: activity in platinum-resistant disease. *J. Clin. Oncol.*, **10**, 243-248.

OMURA, G.A., MORROW, C.P., BLESSING, J.A., MILLER, A., BUCHSBAUM, H.J., HOMESLEY, H.D. & LEONE, L. (1983). A randomised comparison of melphalan versus melphalan plus hexamethylmelamine versus adriamycin plus cyclophosphamide in ovarian carcinoma. *Cancer*, **51**, 783-789.

PERREN, T.J., TAN, S., MATTHEWS, J. & WILTSHAW, E. (1989). Carboplatin in ovarian carcinoma: The Royal Marsden Hospital Experience. In *Proceedings of the Perugia International Cancer Conference II. Recent Advances in the Treatment of Testicular and Ovarian Cancer*. pp. 60-66. LP Communications: New York.

PETO, J. & EASTON, D. (1989). Cancer treatment trials – past failures, current progress and future prospects. *Cancer Surveys*, **8**, 511-533.

PETO, R., PIKE, M.C. & ARMITAGE, P. (1977). Design and analysis of randomised clinical trials requiring prolonged observation of each patient. *Br. J Cancer*, **35**, 1-39.

SCHNITKER, J., BROCK, N., BURKERT, H. & FICHTNER, E. (1976). Evaluation of a cooperative clinical study of the cystostatic agent ifosfamide. *Arzneim-Forsch (Drug Res.)*, **26**, 1783-1793.

SUTTON, G.P., BLESSING, J.A., PHOTOPULOS, G., BERMAN, M.L. & HOMESLEY, H.D. (1990). Gynecologic Oncology Group experience with ifosfamide. *Semin. Oncol.*, **17** (Suppl 4), 6-10.

THIGPEN, J.T., BLESSING, J.A., VANCE, R.B. & LAMBUTH, B.W. (1989). Chemotherapy in ovarian carcinoma: present role and future prospects. *Semin. Oncol.*, **16** (Suppl 6), 58-65.

VAN DYK, J.J., FALKSON, H.C., VAN DER MERWE, A.M. & FALKSON, G. (1972). Unexpected toxicity in patients treated with iphosfamide. *Cancer Res.*, **32**, 921-924.

WILLIAMS, C.J., MEAD, G.M., MACBETH, F.R., THOMPSON, J., WHITEHOUSE, J.M.A., MACDONALD, H., HARVEY, V.J., SLEVIN, M.L., LISTER, T.A., SHEPHERD, J.H. & GOLDING, P. (1985). Cisplatin combination chemotherapy versus chlorambucil in advanced carcinoma: mature results of a randomised trial. *J. Clin. Oncol.*, **3**, 1455-1462.

WILTSHAW, E., SUBRAMARIAN, S., ALEXOPOULOS, C. & BARKER, G.H. (1979). Cancer of the ovary. A summary of experience with cis-dichlorodiammineplatinum (II) at the Royal Marsden Hospital. *Cancer Treat. Rep.*, **63**, 1545-1548.

WILTSHAW, E., EVANS, B., RUSTIN, G., GILBEY, E., BAKER, J. & BARKER, G. (1986). A prospective randomised trial comparing high-dose cisplatin with low-dose cisplatin and chlorambucil in advanced ovarian carcinoma. *J. Clin. Oncol.*, **4**, 722-729.

WILTSHAW, E. & KRONER, T. (1976). Phase II study of cis-dichlorodiammineplatinum (II) (NSC-119875) in advanced adenocarcinoma of the ovary. *Cancer Treat. Rep.*, **60**, 55-60.

WORLD HEALTH ORGANISATION (1922). WHO handbook for reporting results of cancer treatment. Offset publication no. 48. *WHO*, WHO: Geneva.